# Supplementary material for: Evolving affinity between Coulombic reversibility and hysteretic phase transformations in nano-structured silicon-based lithium-ion batteries
Source: Nat Commun. 2018 Feb 2;9:479. doi: 10.1038/s41467-018-02824-w (PMC5797158; doi:10.1038/s41467-018-02824-w)
Supplement: Supplementary file 1 — Supplementary Information [file 41467_2018_2824_MOESM1_ESM.pdf]

Supplementary information (SI)

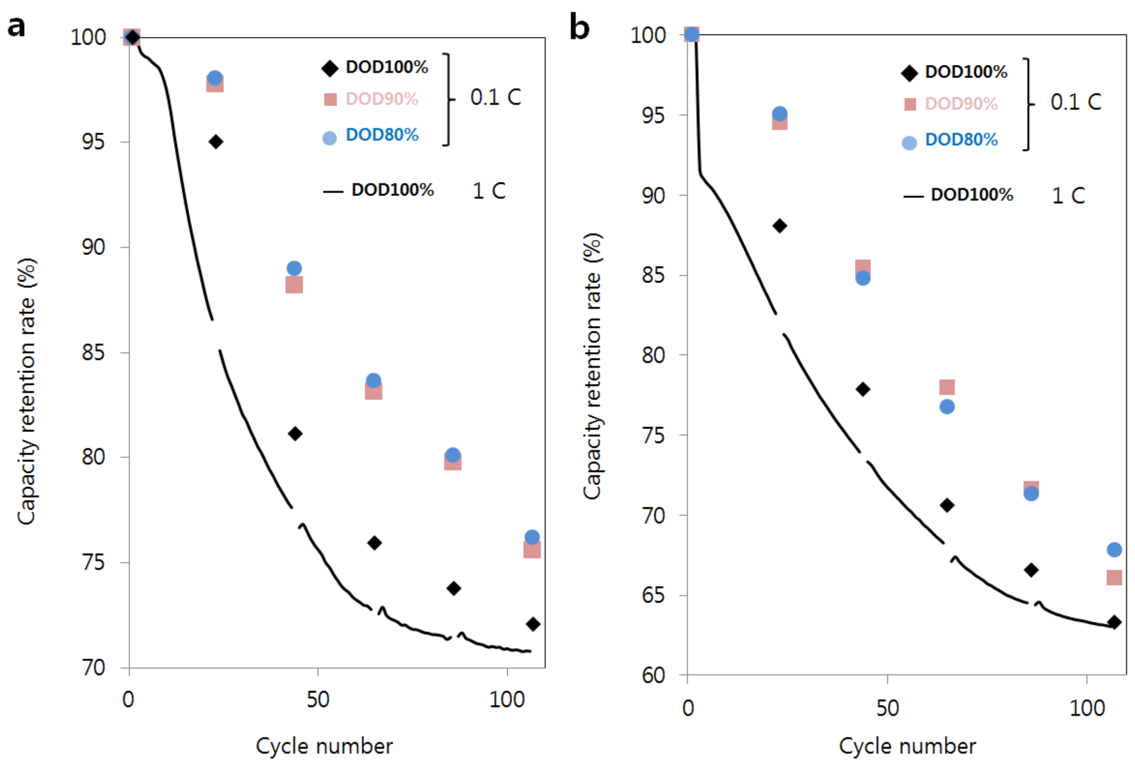

Supplementary Fig. 1

Capacity retention rate for different depth of discharge (DOD). Capacity retention rate over 107 cycles for (**a**) type-A and (**b**) type-B electrodes in 2032-type coin half-cells. The solid dots show the retention at the probing points. Note that at the probing points (inserted every 20 cycles), all the electrodes are cycled at 0.1 C regardless of the DOD cycling history. Black, pink, and blue dots correspond to the retentions under DOD100%, 90%, and 80% cycling protocols, respectively. The black solid lines show control retention profiles under DOD100% at 1 C.

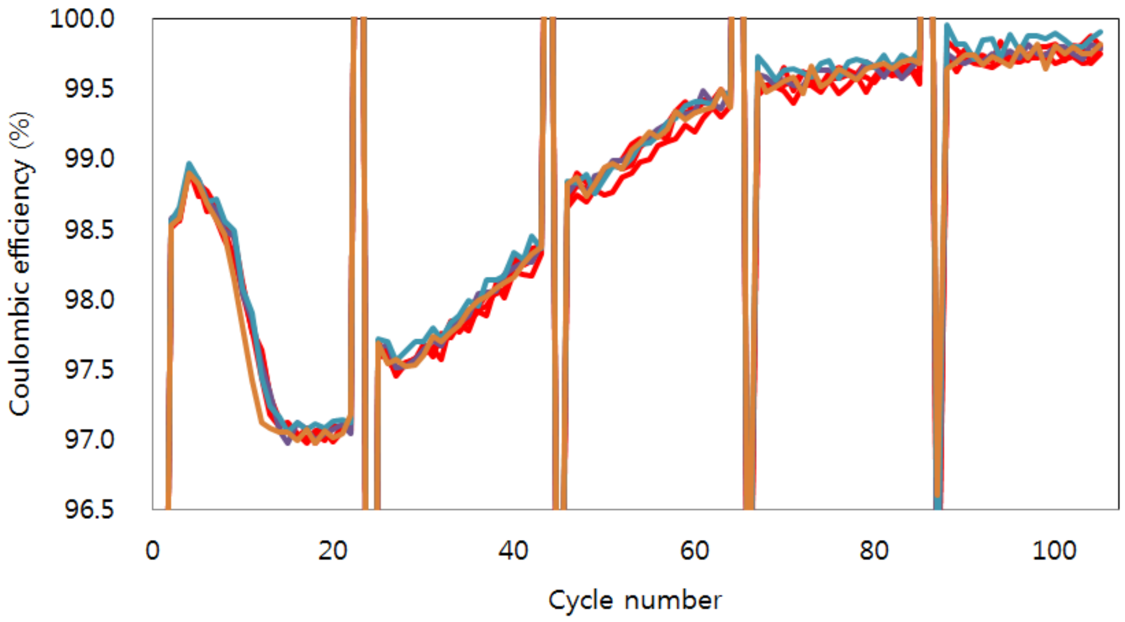

Supplementary Fig. 2

Accuracy and reproducibility tests for Coulombic efficiency (CE) for six identically prepared type-A electrodes in 2032-type coin half-cells cycled under depth of discharge (DOD)100% at 1 C. Standard deviation of the profiles is estimated to be ~0.07% (see Methods > 'Accuracy of cycler' for details).

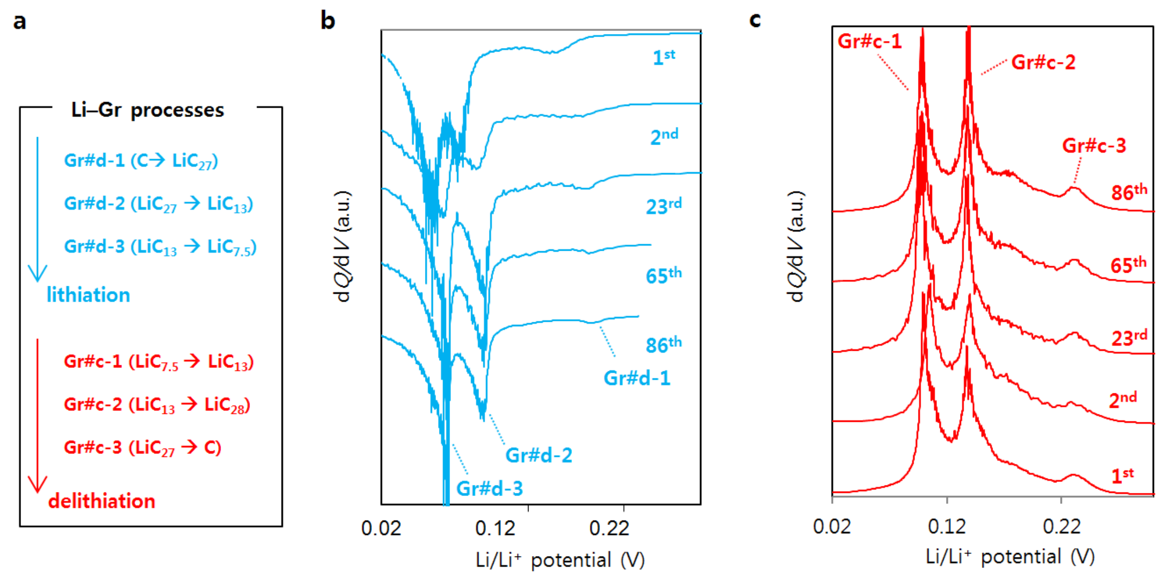

Supplementary Fig. 3

Electrochemical Li–Graphite (Gr) processes for bare Gr electrodes in 2032-type coin half-cells at the probing points under depth of discharge (DOD)100%. Note that at the probing points (inserted every 20 cycles), the electrodes are cycled at 0.1 C while at the other cycling points cycled at 1 C. **a** Schematics showing electrochemical Li–Gr processes, which are denoted as Gr#d-X/Gr#c-X, for the Xth discharge/charge processes, respectively. dQ/dV profiles on (**b**) lithiation and (**c**) delithiation at the probing points. The profiles are stacked with a constant pitch to show the different processes more clearly.

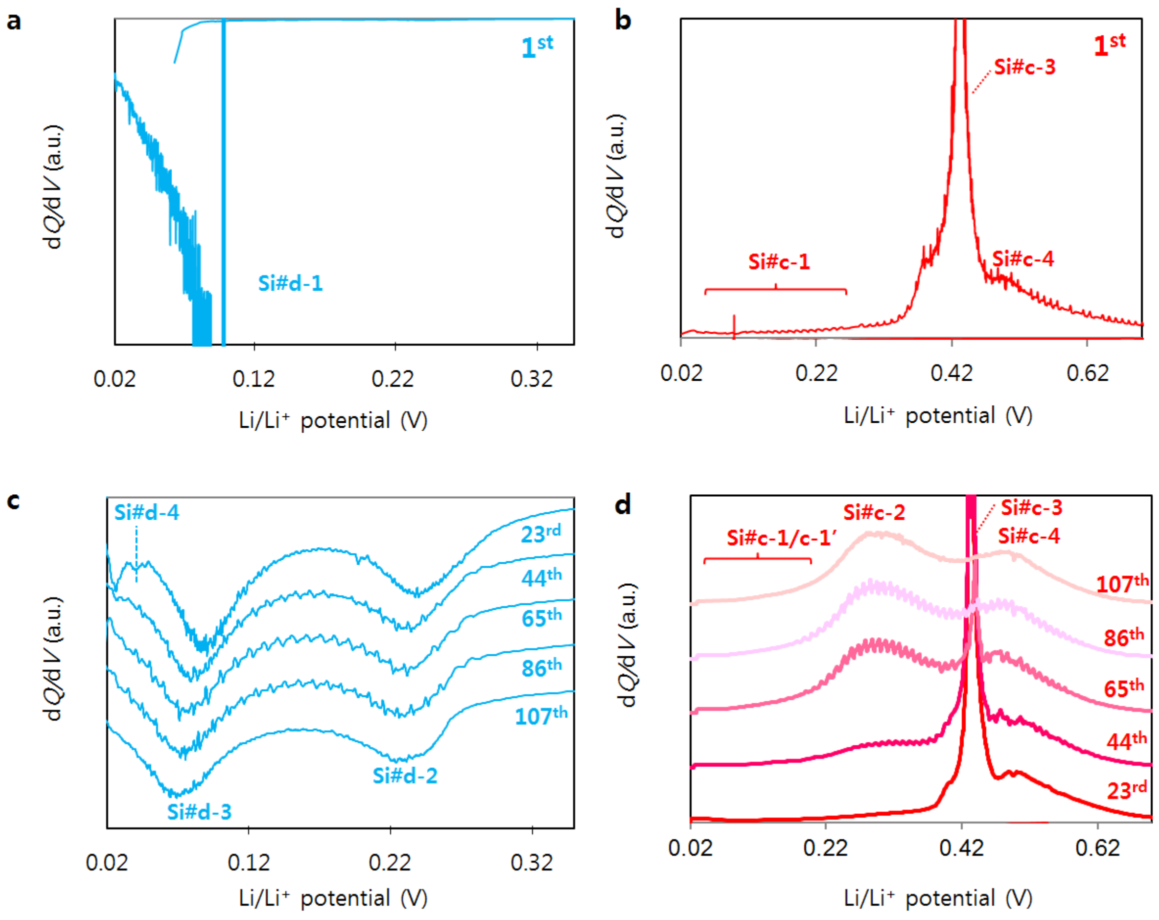

Supplementary Fig. 4

dQ/dV profiles of type-B electrodes in 2032-type coin half-cells. Electrodes are cycled under depth of discharge (DOD)100%. dQ/dV profiles in the initial amorphization processes of polycrystalline Si nanoparticles on (**a**) lithiation and (**b**) delithiation at 0.1 C. The profiles at the probing points in the following cycles on (**c**) lithiation and (**d**) delithiation. Note that at the probing points (inserted every 20 cycles), the electrodes are cycled at 0.1 C while at the other cycling points cycled at 1 C. The different electrochemical Li–Si processes are labelled as Si#d-X/Si#c-X for the Xth discharge/charge process, respectively. The dQ/dV profiles are stacked with a constant pitch to show the different processes more clearly.

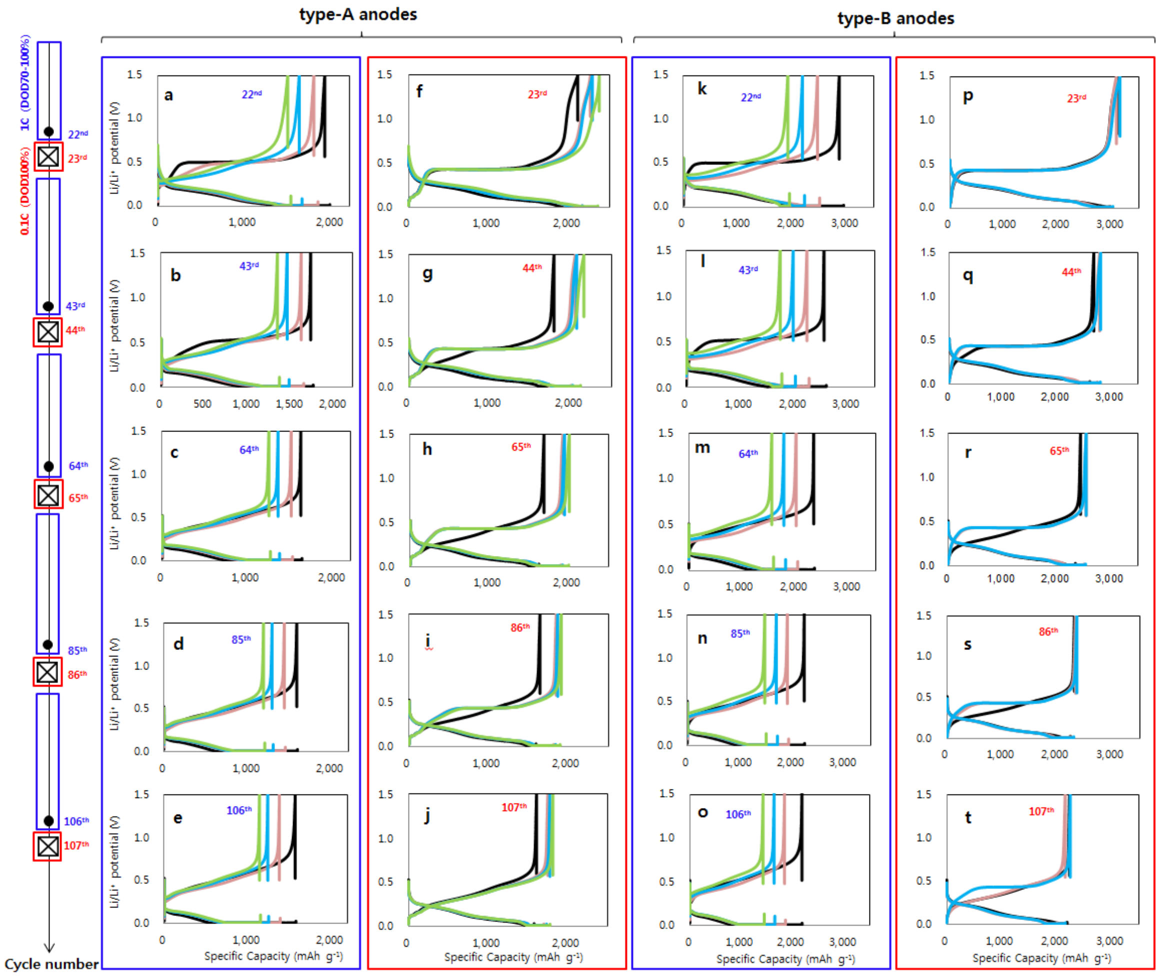

Supplementary Fig. 5

Li/Li$^+$ potential in 2032-type coin half-cells as a function of the specific capacity, for type-A (left two lines) and -B electrodes (right two lines) at the cycle previous to the probing point (surrounded by blue rectangle) and the probing points (surrounded by red rectangle) . Note that at the probing points (inserted every 20 cycles), all the electrodes are cycled at 0.1 C regardless of the depth of discharge (DOD) cycling history while at the other cycling points cycled at 1 C under the given DOD controls. **a**–**e** The cycles previous to the probing points for type-A electrodes, (**f**–**j**) the probing points for type-A electrodes, (**k**–**o**) the cycles previous to the probing points for type-B electrodes, and (**p**–**t**) the probing points for type-B electrodes. Black, pink, blue, and green profiles correspond to cycling under DOD100%, 90%, 80%, and 70%, respectively.

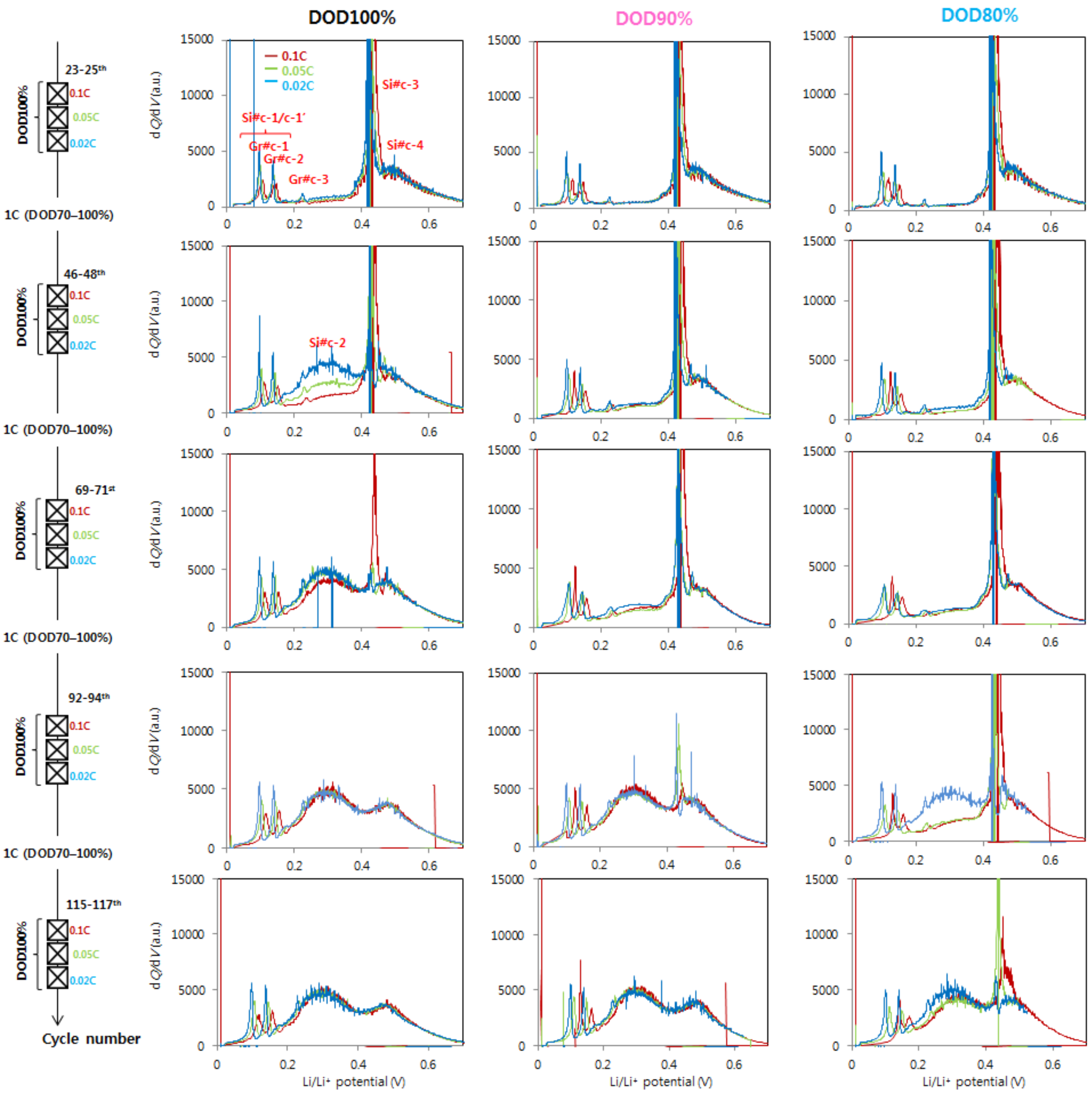

Supplementary Fig. 6

Dependence of Li–Si processes on current rates at probing points cycled under depth of discharge (DOD)100% with different DOD history for type-A electrodes in 2032-type coin half-cells; Evolution of Si#c-1–3 processes on delithiation at the probing points is shown by d*Q*/d*V* profiles. Note that at the probing points (inserted every 20 cycles), all the electrodes are cycled at slower current rates regardless of the DOD cycling history while at the other cycling points cycled at 1 C under the given DOD controls. The Li–Si processes (Si#d-X/Si#c-X represents the Xth Li–Si discharge/charge process) are summarized in Fig. 3a and Supplementary Table 5 (also see Methods > 'Reference electrochemistry'). At the probing points, three lower current rates (0.02, 0.05, and 0.1 C) are used consecutively to observe the rate dependence of the processes, shown as blue, green, and red profiles, respectively.

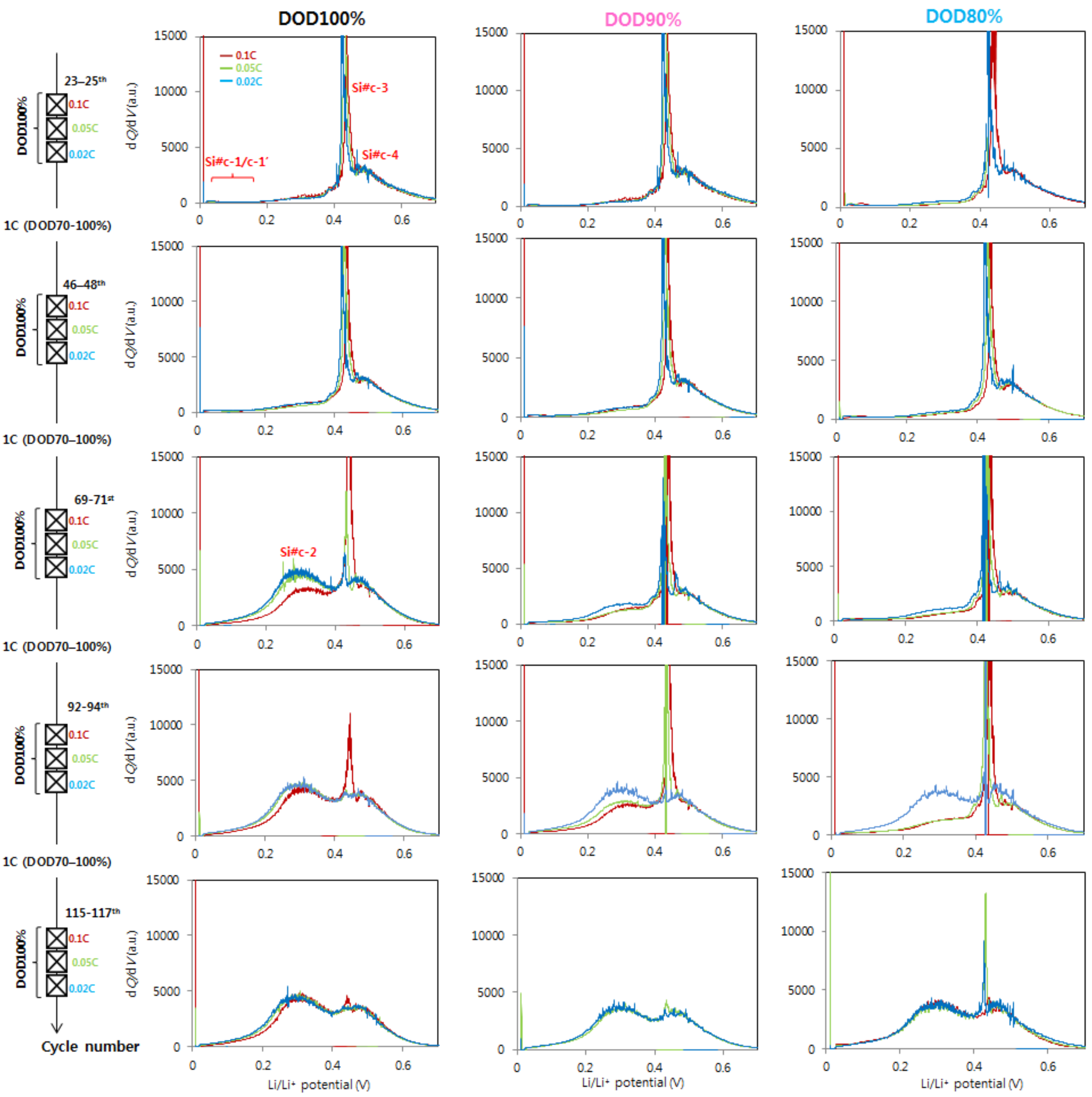

Supplementary Fig. 7

Dependence of Li–Si processes on current rates at probing points cycled under depth of discharge (DOD)100% with different DOD history for type-B electrodes in 2032-type coin half-cells; Evolution of Si#c-1–3 processes on delithiation at the probing points is shown by d*Q*/d*V* profiles. Note that at the probing points (inserted every 20 cycles), all the electrodes are cycled at slower current rates regardless of the DOD cycling history while at the other cycling points cycled at 1 C under the given DOD controls. The Li–Si processes (Si#d-X/Si#c-X represents the Xth Li–Si discharge/charge process) are summarized in Fig. 3a and Supplementary Table 5 (also see Methods > 'Reference electrochemistry'). At the probing points, three lower current rates (0.02, 0.05, and 0.1 C) are used consecutively to observe the rate dependence of the processes, shown as blue, green, and red profiles, respectively.

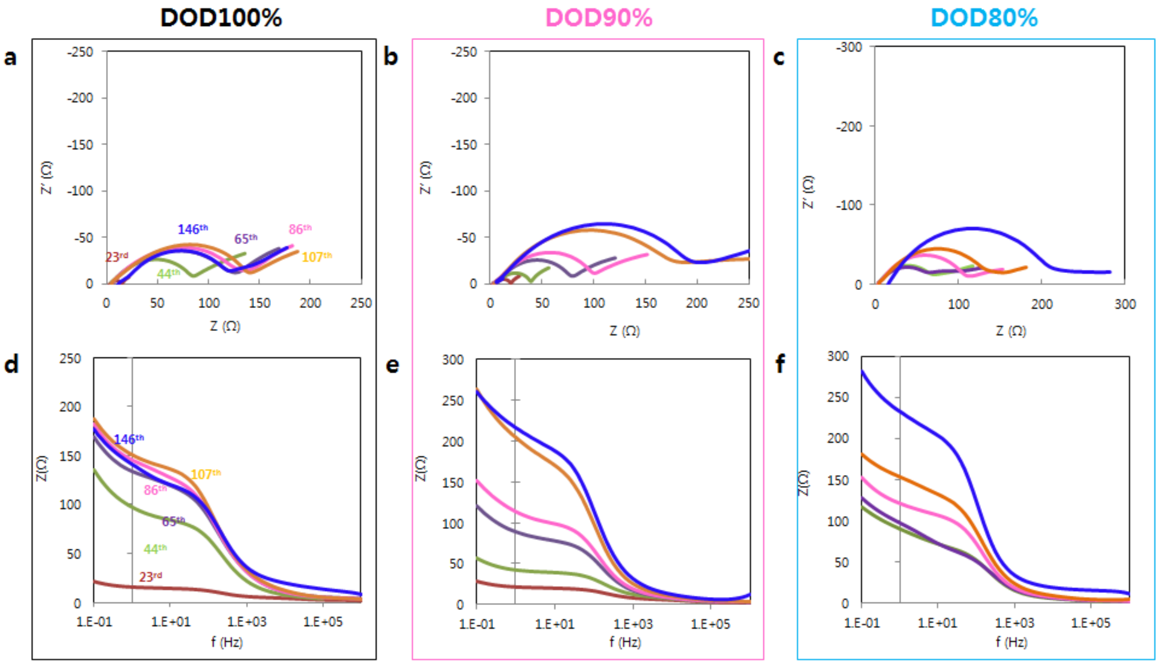

Supplementary Fig. 8

Electrochemical impedance spectroscopy (EIS) data for fully lithiated type-A electrodes in 2032-type coin half-cells cycled under depth of discharge (DOD)80–100% over ~150 cycles. The cell is cycled at 1 C under the subjected DOD controls until it reaches the cycle just prior to the probing point (inserted every 20 cycles). Then, the cell is cycled at 0.05 C, holding the voltage at 10 mV on lithiation for at least 24 h until the residual current becomes less than 0.001 C. The EIS frequency is swept from 1 MHz to 0.1 Hz with a fluctuating voltage of ±5 mV. **a–c** Impedance component of a real part plotted over an imaginary part for DOD100, 90, and 80%, respectively. **d–f** Measurement frequency plotted over impedance component of a real part for DOD100, 90, and 80%. These profiles under DOD100, 90, and 80% are encircled by black, pink, and blue rectangles, respectively. Red, green, purple, pink, orange, and blue lines correspond to the profiles at the 23rd, 44th, 65th, 86th, 107th, and 146th cycle, respectively.

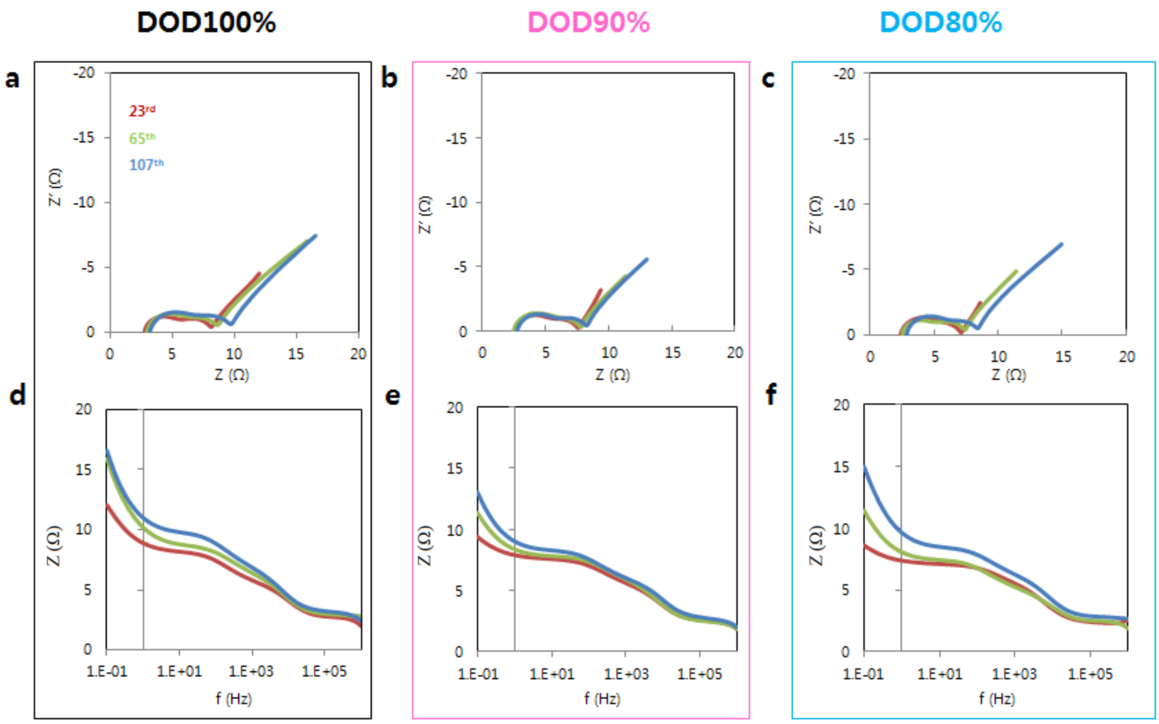

Supplementary Fig. 9

Electrochemical impedance spectroscopy (EIS) data for fully lithiated type-A electrodes in 2032-type symmetric cells cycled under depth of discharge (DOD) 80–100%. Two identical cells are cycled at 1 C under the subjected DOD protocols, until they reach the cycle just prior to the probing point (inserted every 20 cycles). Then, the cell is cycled at 0.05 C, holding the voltage at 10 mV on lithiation for at least 24 h until the residual current becomes less than 0.001 C. The two coin cells are then disassembled in Ar-filled glovebox and re-assembled into one symmetric coin cell with new electrolyte and separator. The EIS frequency is swept from 1 MHz to 0.1 Hz with a fluctuating voltage of ±5 mV. **a–c** Impedance component of a real part plotted over an imaginary part for DOD100, 90, and 80%, respectively. **d–f** Measurement frequency plotted over impedance component of a real part for DOD100, 90, and 80%. These profiles under DOD100, 90, and 80% are encircled by black, pink, and blue, respectively. Red, green, and blue lines correspond to the profiles at the 23rd, 65th, and 107th cycle, respectively.

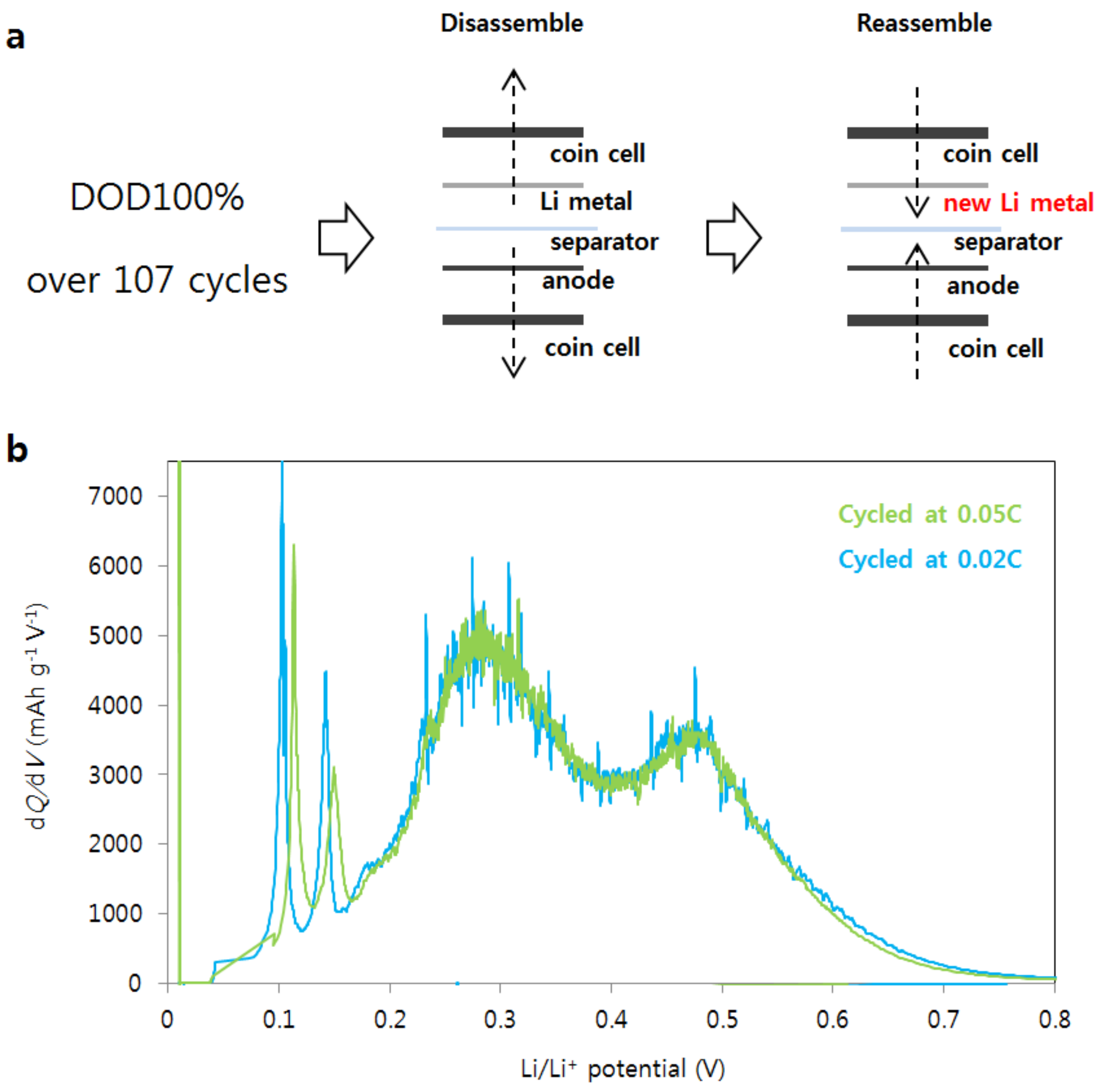

Supplementary Fig. 10

Examination of Li-metal resistivity effect in half coin-cells, accumulated over cycles, on Li–Si electrochemical processes. **a** Schematics of the experimental flow; Type-A electrode is cycled in 2032-type coin half-cell for 107 cycles under DOD100% protocol. Afterwards, the cell is disassembled in Ar-filled glovebox and reassembled with fresh, polished Li-metal with new electrolyte. **b** d*Q*/d*V* profiles on delithiation of type-A electrodes at 0.05 C (green) and 0.02 C (blue) in the reassembled 2032-type half-cell. Green and blue profiles correspond to cycling at 0.05 and 0.02 C, respectively. The sharp peak at 430 mV (Si#c-3, a signature of c-$Li_{3.75}Si$ decomposition into a-$Li_{<\sim1.1}Si$) is absent, and instead the Li–Si processes are dominated by Si#c-2 and Si#c-4. For definition of the Li–Si processes see Fig. 3a (also see Methods > 'Reference electrochemistry').

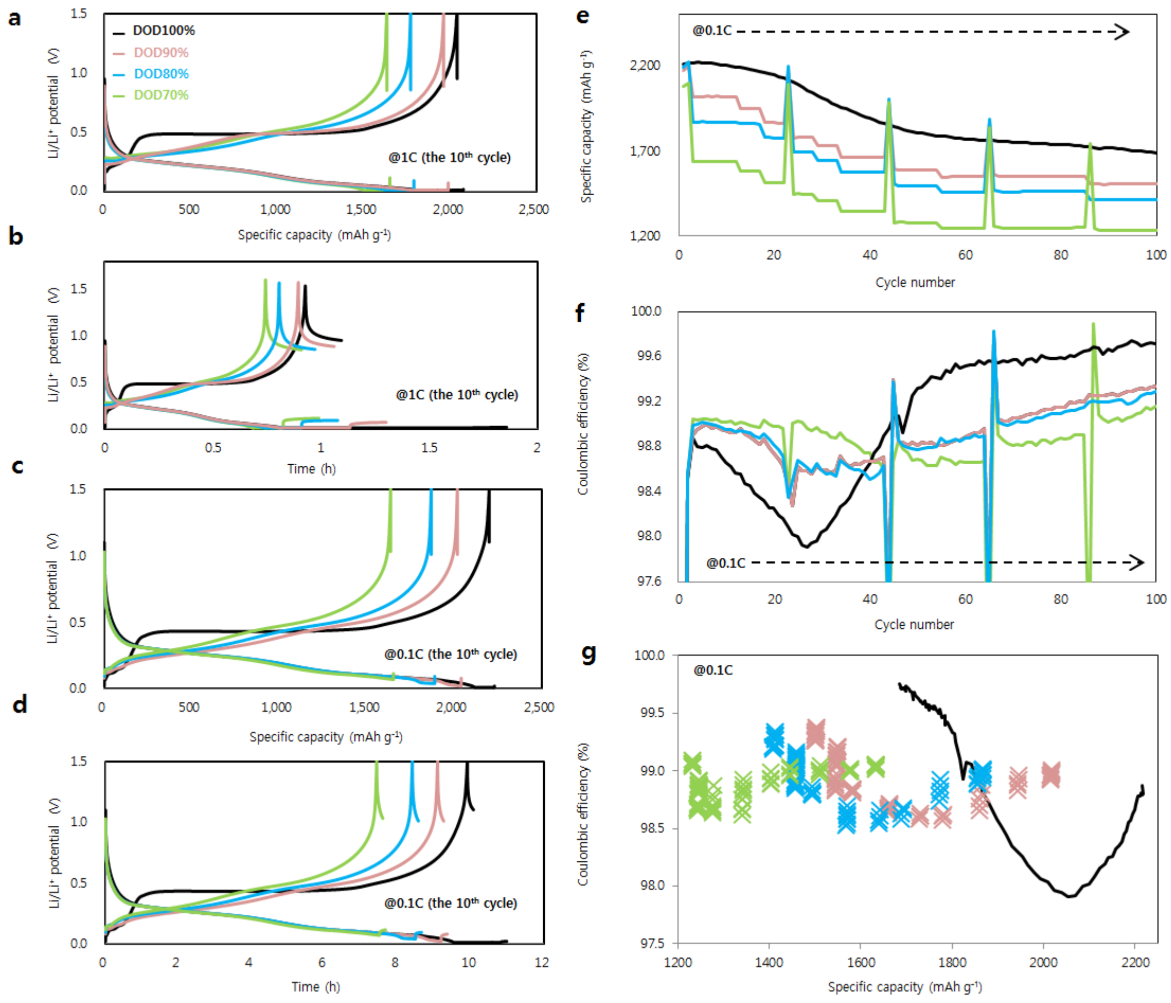

Supplementary Fig. 11

Effect of electrode/electrolyte exposure time at constant voltage (CV) domain (10 mV) on Coulombic efficiency (CE) for type-A electrodes. Li/Li^+ potential in 2032-type coin half-cells as a function of (**a**, **c**) specific capacity (mAh g^{-1}) and (**b**, **d**) cycling time in the 10th cycle at a current rates of (**a**, **b**) 1 C and (**c**, **d**) 0.1 C. The total duration of lithiation is not proportional to the depth of discharge (DOD) percentages at 1 C, due to the nature of the constant current constant voltage (CCCV) mode, while they are proportional to each other at 0.1 C. **e** Specific capacity and (**f**) CE cycled at 0.1 C with different DOD controls. **g** CE as a function of specific capacity cycled at 0.1 C under different DOD controls. Black solid line corresponds to DOD100%, and pink, blue, and green profiles/dots correspond to DOD90%, 80%, and 70%, respectively. CE error bars are all within a range of ±0.1% and omitted in the figure.

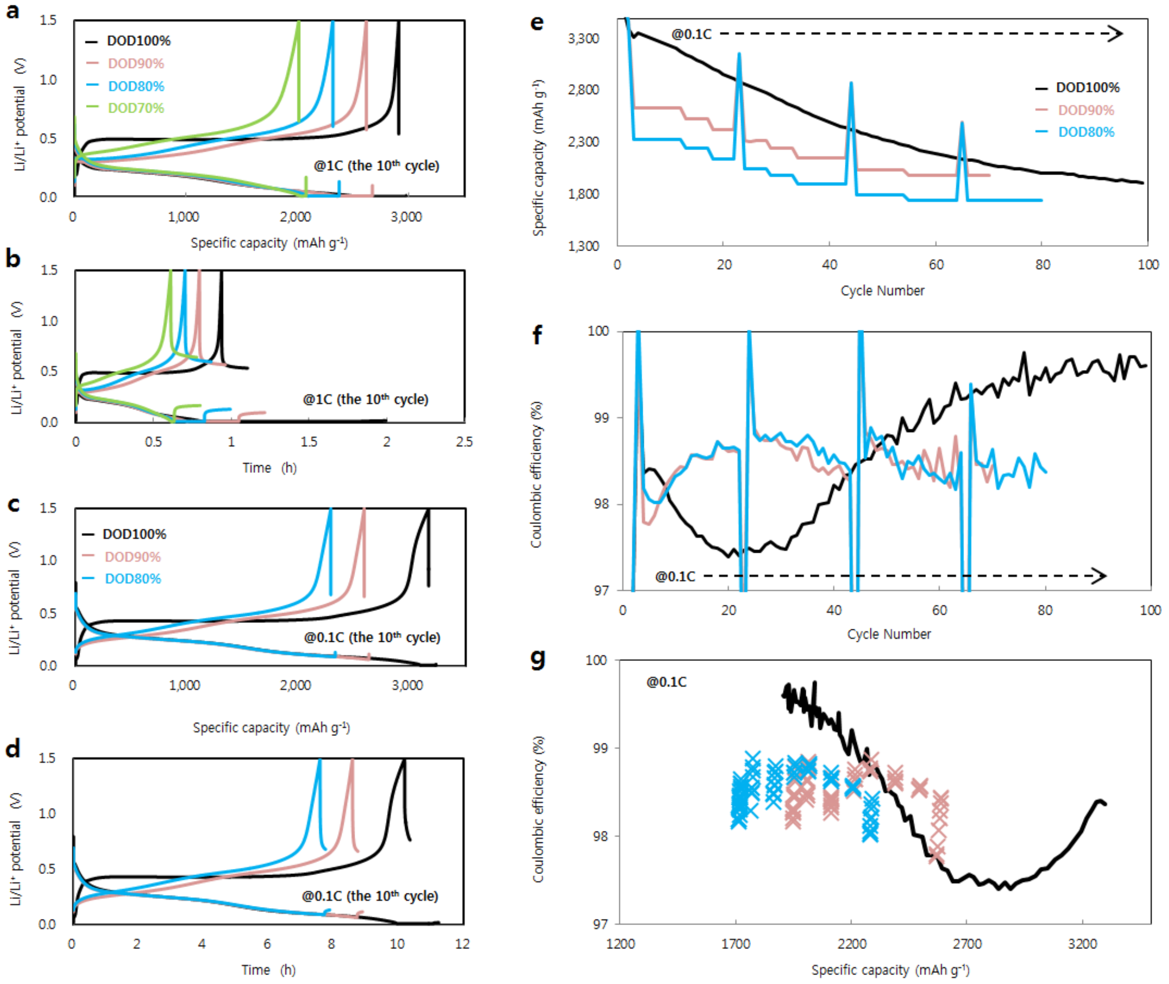

Supplementary Fig. 12

Effect of electrode/electrolyte exposure time at constant voltage (CV) domain (10 mV) on Coulombic efficiency (CE) for type-B electrodes. Li/Li^+ potential in 2032-type coin half-cells as a function of (**a**, **c**) specific capacity (mAh g^{-1}) and (**b**, **d**) cycling time in the 10th cycle at a current rate of (**a**, **b**) 1 C and (**c**, **d**) 0.1 C. The total duration of lithiation is not proportional to the depth of discharge (DOD) percentages at 1 C, due to the nature of the constant current constant voltage (CCCV) mode, while they are proportional to each other at 0.1 C. **e** Specific capacity and (**f**) CE cycled at 0.1 C with different DOD controls. **g** CE as a function of specific capacity cycled at 0.1 C under different DOD controls. Black solid line corresponds to DOD100%, and pink and blue profiles/dots correspond to DOD90% and 80%, respectively. CE error bars are all within a range of ±0.1% and omitted in the figure.

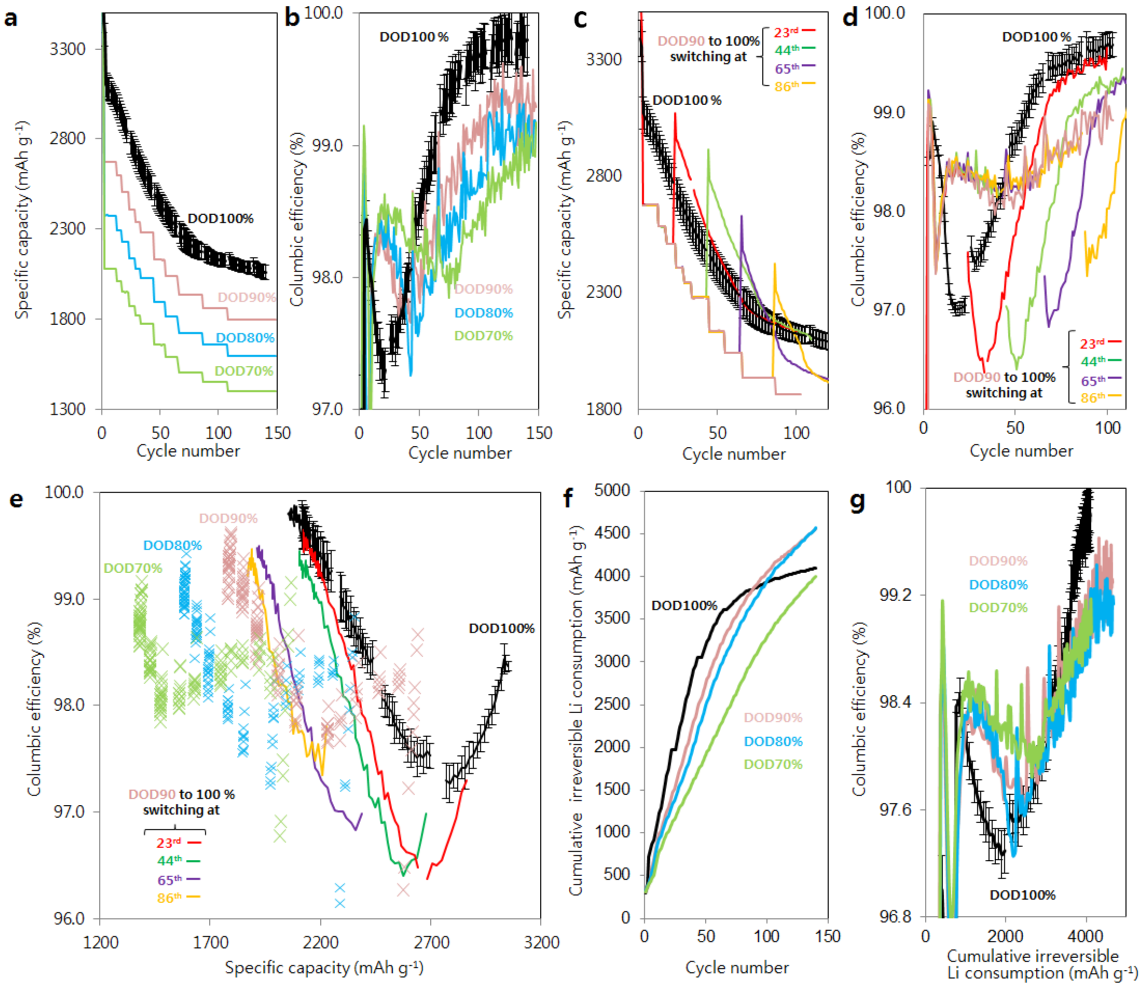

Supplementary Fig. 13

Electrochemical reversibility outputs for type-B electrode. **a** Specific capacity and (**b**) Coulombic efficiency (CE) under different depth of discharge (DOD) over 150 cycles. **c** Specific capacity and (**d**) CE for DOD90%, switched to DOD100% at different cycle numbers. The switching points for red, green, purple, and yellow solid lines are the 23rd, 44th, 65th, and 86th, respectively. **e** CE as a function of the specific capacity for different DOD protocols. Coloured solid lines (red, green, purple, and yellow) show the profiles switched from DOD90% to DOD100% in (**c**, **d**). It is clear that CE under DOD100% is the highest among all cases, after a certain loss of reversible capacity. **f** Cycle number plotted over accumulated irreversible Li consumption. The cumulative consumption under DOD100% is the lowest for the given DODs after certain cycles. **g** CE plotted over accumulated irreversible Li consumption. CE reaches under DOD100% protocol is the highest among the given DOD controls after a certain amount of sacrificial Li consumption. Note that error bars of CE for DOD70–90% are all within a range of ±0.1% and therefore omitted in (**b**, **d**, **e**, **g**) (see Methods > 'Accuracy of cycler').

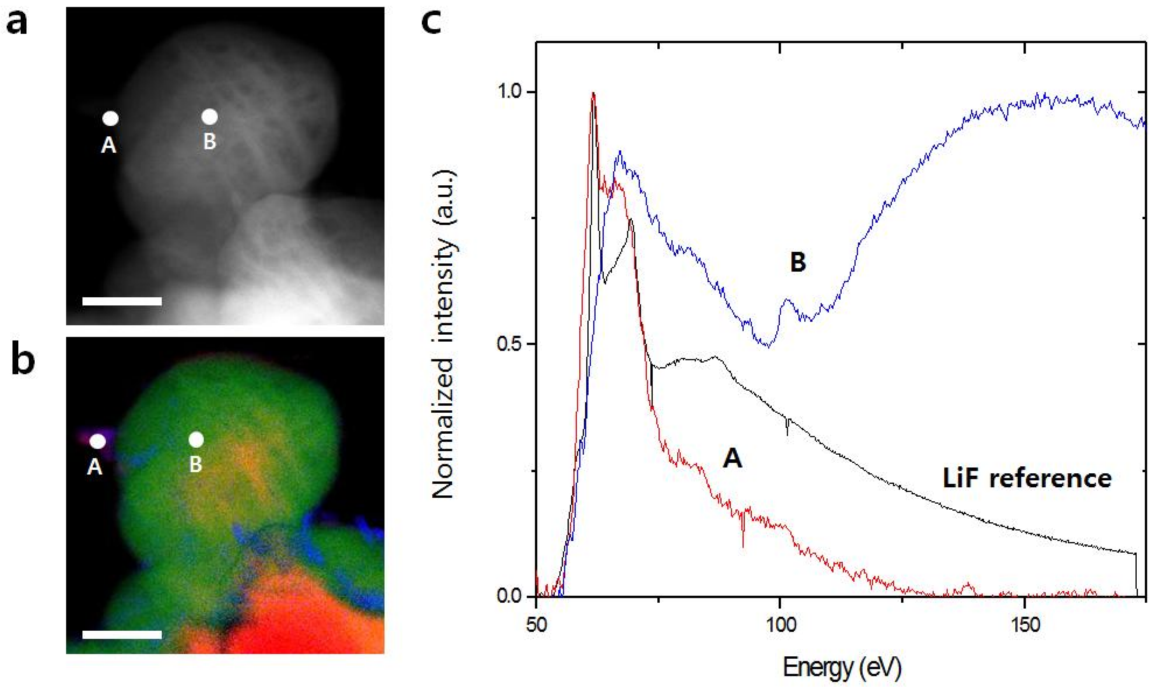

Supplementary Fig. 14

Electron microscopy image and electron energy loss spectroscopy (EELS) spot analysis of a delithiated type-A electrode after the first two cycles under depth of discharge (DOD)100%. Polycrystalline Si nanoparticles are fully amorphized and become porous nanostructures. **a** High-angle annular dark field (HAADF) image. **b** Overlapped EELS spectrum image (SI) elemental mapping, with green, blue, and red represent Si, C, and Li, respectively. All the scale bars are 50 nm. **c** EELS spectra for the two spots shown in (**a**, **b**), indicating that LiF is concentrated near the surface of the spherical particle.

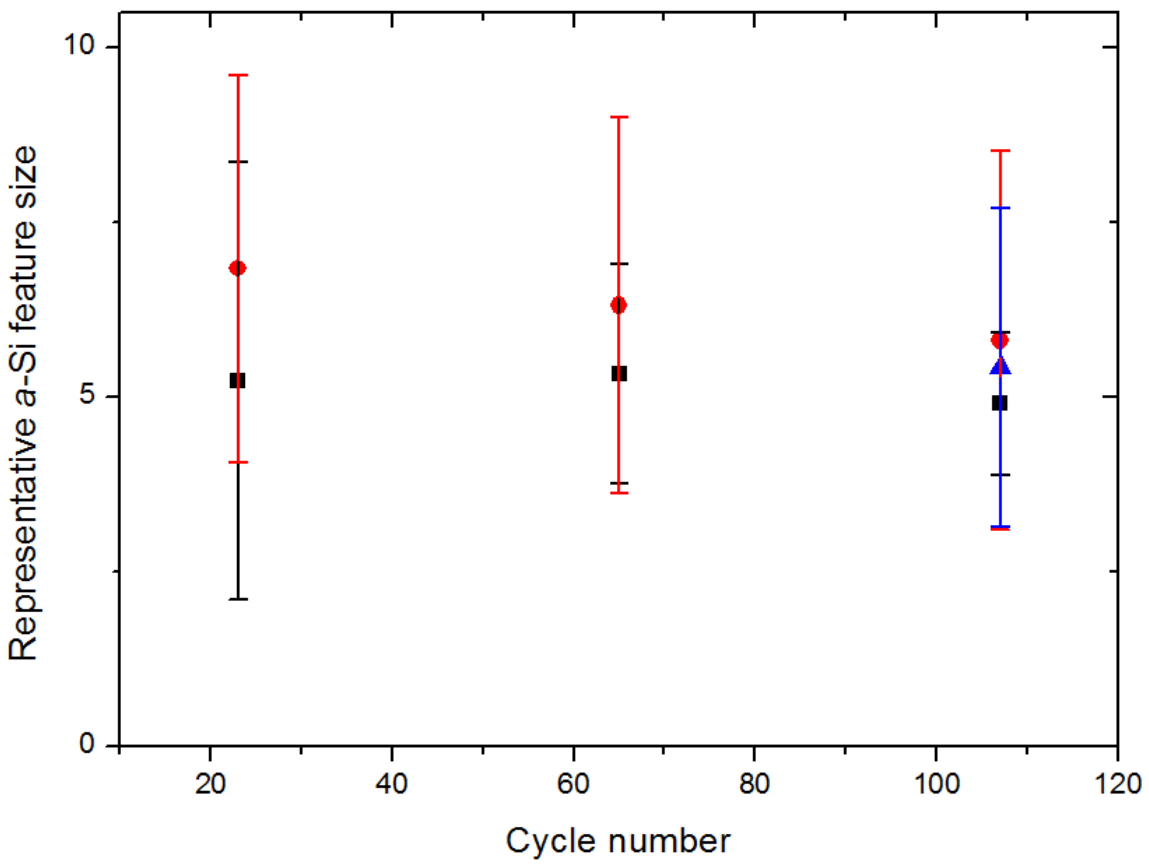

Supplementary Fig. 15

Mean feature size of representative amorphous Si structures at various cycle numbers for type-A electrodes cycled in 2032-type coin half-cells under depth of discharge (DOD) 80–100%. The size is determined from 50–100 randomly picked spots in the TEM images of fully delithiated electrodes. The error bars show the standard deviations. Black, red, and blue data points correspond to DOD100%, 90%, and 80%, respectively.

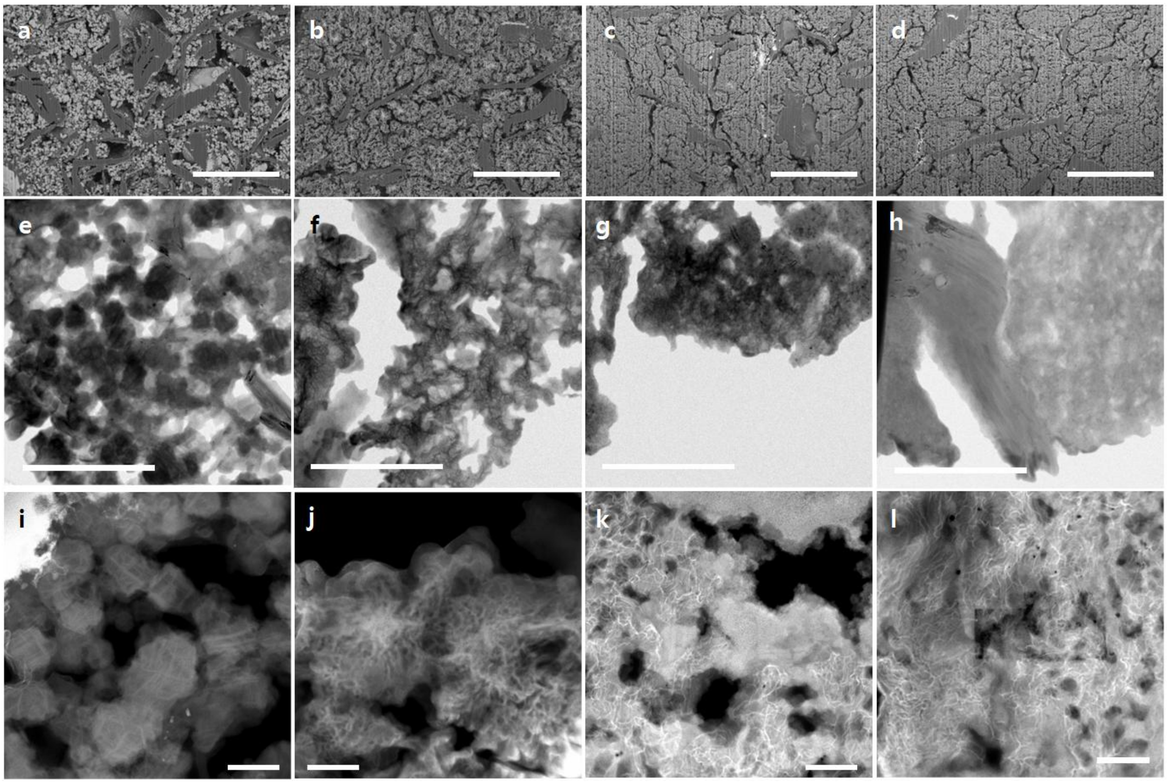

Supplementary Fig. 16

Electron microscopy images of fully delithiated type-A electrodes over cycles in 2032-type coin half-cells under depth of discharge (DOD)100% protocol. **a**–**d** Back scattering electron (BSE) images of electrode cross sections sliced via focused ion beam (FIB), (**e**–**h**) bright-field (BF) TEM images, and (**i**–**l**) high-angle annular dark field (HAADF) images of the sliced electrodes for (**a**, **e**, **i**) the 3rd (after the amorphization of polycrystalline Si nanoparticles), (**b**, **f**, **j**) the 23rd, (**c**, **g**, **k**) the 65th, and (**d**, **h**, **l**) the 107th cycle (see details in Methods under 'TEM'). Prior to the observation, the coin cell is cycled at 1 C to reach the target number of cycles, being fully delithiated at 1.5 V. The electrodes are then washed with dimethyl carbonate (DMC) in Ar-filled glovebox, vacuum dried, and transferred to the TEM holder via airtight transfer vessel without exposure to the ambient air. The scale bars in (**a**–**d**), (**e**–**h**), and (**i**–**l**) are 5 μm, 1 μm, and 200 nm, respectively.

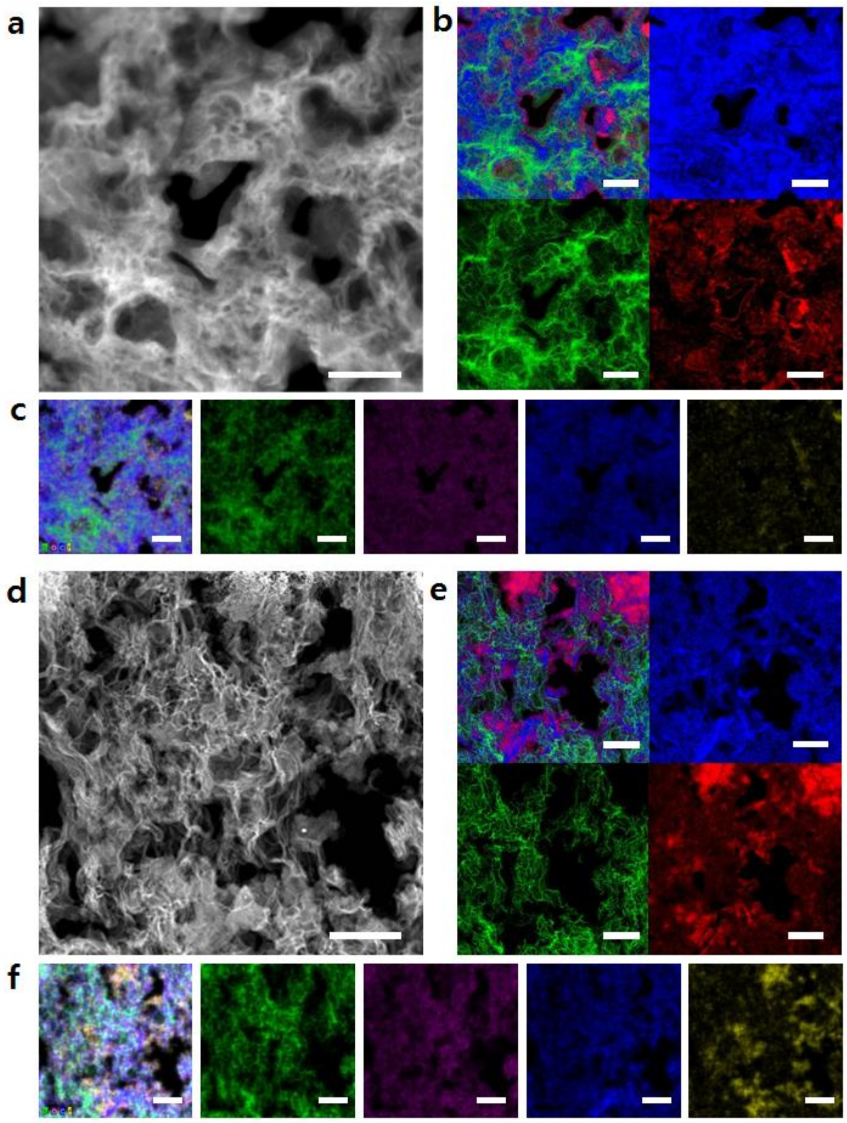

Supplementary Fig. 17

Elemental mapping for delithiated type-A electrode over cycles under depth of discharge (DOD)100%. **a**, **d** High-angle annular dark field (HAADF) images, (**b**, **e**) electron energy loss spectroscopy (EELS) spectrum image (SI) elemental mapping, and (**c**, **f**) energy dispersive X-ray spectroscopy (EDS) data of delithiated type-A electrodes. 2032-Type coin half-cells are cycled under DOD100% for (**a**–**c**) 23 and (**d**–**f**) 65 cycles. In the EELS SI elemental mapping, blue, green, and red correspond to C, Si, and Li, respectively. In the EDS elemental mapping, green, purple, blue, and yellow show Si, O, C, and F, respectively. Prior to the observation, the coin half-cell is cycled at 1C to the target cycle number and fully delithiated at 1.5 V. The electrodes are then washed with dimethyl carbonate (DMC) in Ar-filled glovebox, vacuum dried, and transferred to the TEM holder via in-house airtight vessel (see details in Methods under 'TEM'). The scale bars are all 200 nm.

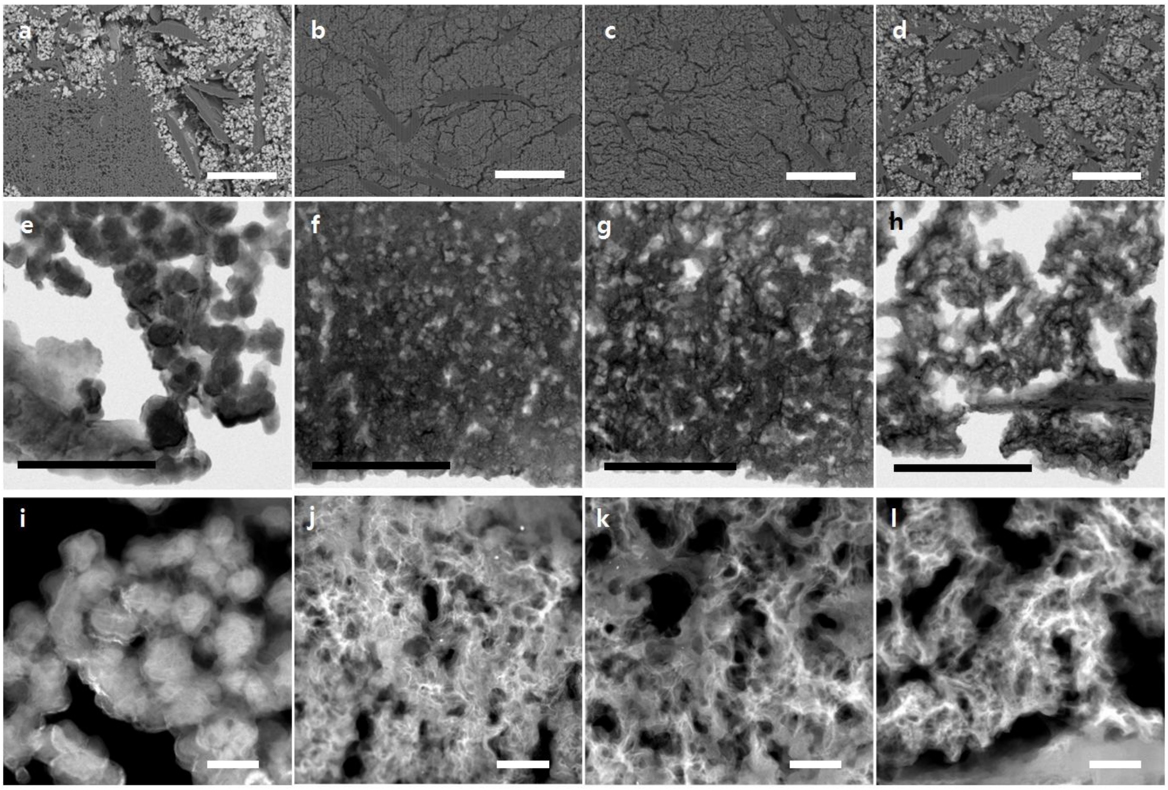

Supplementary Fig. 18

Electron microscopy images of fully delithiated type-A electrodes over cycles in 2032-type coin half-cells under depth of discharge (DOD)90% protocol. **a**–**d** Back scattering electron (BSE) images of electrode cross sections sliced via focused ion beam (FIB), (**e**–**h**) bright-field (BF) TEM images, and (**i**–**l**) high-angle annular dark field (HAADF) images of the sliced electrodes for (**a**, **e**, **i**) the 3rd (after the amorphization of polycrystalline Si nanoparticles), (**b**, **f**, **j**) the 23rd, (**c**, **g**, **k**) the 65th, and (**d**, **h**, **l**) the 107th cycle (see details in Methods under 'TEM'). Prior to the observation, the coin cell is cycled at 1 C to reach the target number of cycles, being fully delithiated at 1.5 V. The electrodes are then washed with dimethyl carbonate (DMC) in Ar-filled glovebox, vacuum dried, and transferred to the TEM holder via airtight transfer vessel without exposure to the ambient air. The scale bars in (**a**–**d**), (**e**–**h**), and (**i**–**l**) are 5 µm, 1 µm, and 200 nm, respectively.

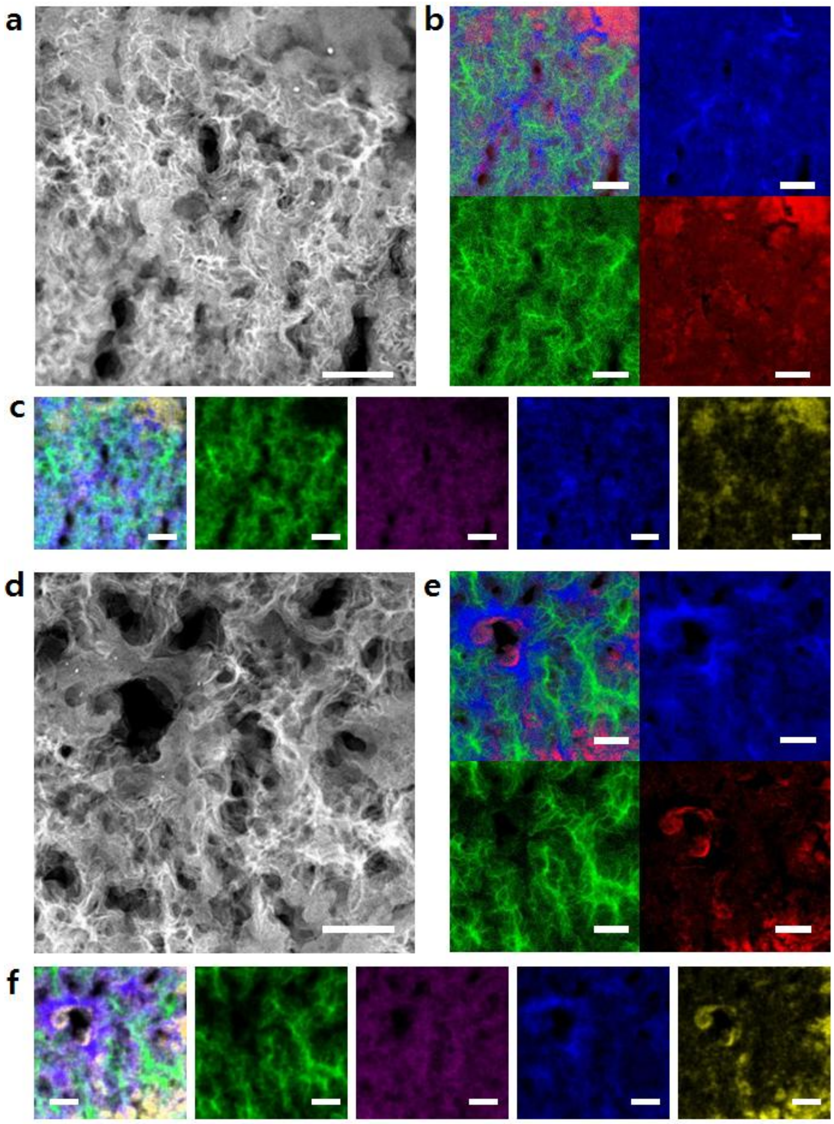

Supplementary Fig. 19

Elemental mapping for delithiated type-A electrode over cycles under depth of discharge (DOD)90%. **a**, **d** High-angle annular dark field (HAADF) images, (**b**, **e**) electron energy loss spectroscopy (EELS) spectrum image (SI) elemental mapping, and (**c**, **f**) energy dispersive X-ray spectroscopy (EDS) data of delithiated type-A electrodes. 2032-Type coin half-cells are cycled under DOD100% for (**a**–**c**) 23 and (**d**–**f**) 65 cycles. In the EELS SI elemental mapping, blue, green, and red correspond to C, Si, and Li, respectively. In the EDS elemental mapping, green, purple, blue, and yellow show Si, O, C, and F, respectively. Prior to the observation, the coin half-cell is cycled at 1C to the target cycle number and fully delithiated at 1.5 V. The electrodes are then washed with dimethyl carbonate (DMC) in Ar-filled glovebox, vacuum dried, and transferred to the TEM holder via in-house airtight vessel (see details in Methods under 'TEM'). The scale bars are all 200 nm.

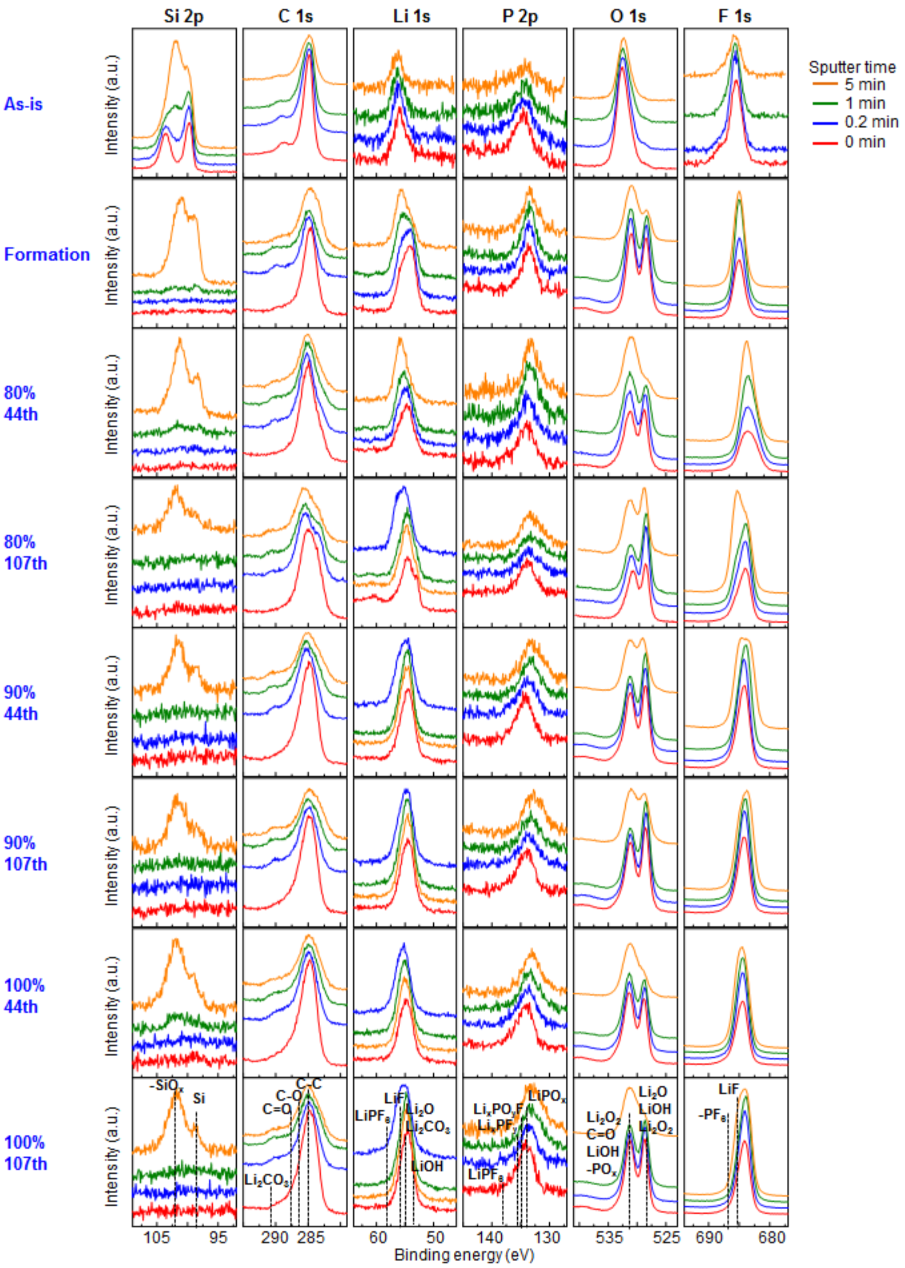

Supplementary Fig. 20

Si-2p, C-1s, Li-1s, P-2p, O-1s, and F-1s XPS spectra with different ion beam sputtering times for type-A electrodes before cycling (the 1st row), after the amorphization of polycrystalline Si nanoparticles (the 2nd row), and delithiated electrodes after the 44th and 107th cycles under depth of discharge (DOD) 80–100% (the 3rd–8th rows). Prior to the observation, the coin cells are fully delithiated, holding the potential at 1.5 V until the current decays to less than 0.001 C. The electrodes are then washed using dimethyl carbonate (DMC) in Ar-filled glovebox, vacuum dried, and transferred to in-house XPS holder without exposure to the ambient air (see details in Methods > 'XPS').

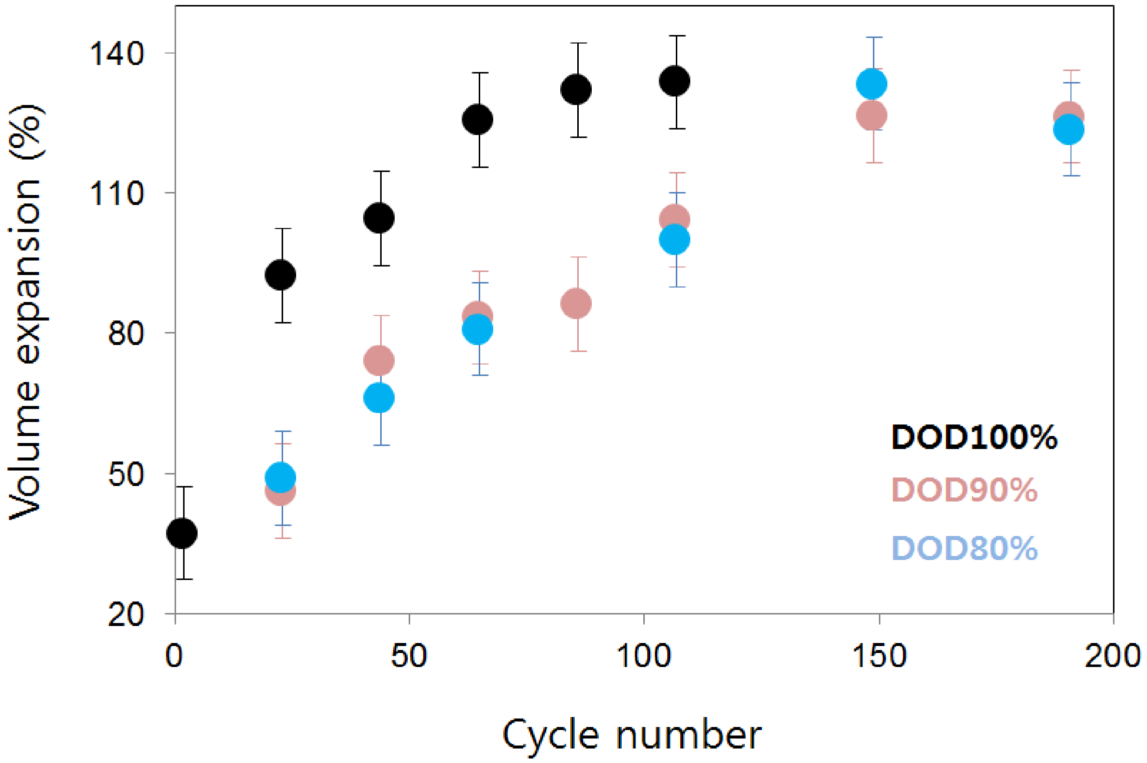

Supplementary Fig. 21

Ex situ measured volume expansion rate for fully delithiated type-A electrodes at the probing points cycled under depth of discharge (DOD)80–100% controls; the value is tabulated from electrode thickness before/after the cycles. Black, pink, and blue dots correspond to the expansion rate under DOD100%, 90%, and 80% cycling protocols, respectively. The electrode thickness is measured by a commercial micrometer (Mitsutoyo) with an accuracy of 1 µm.

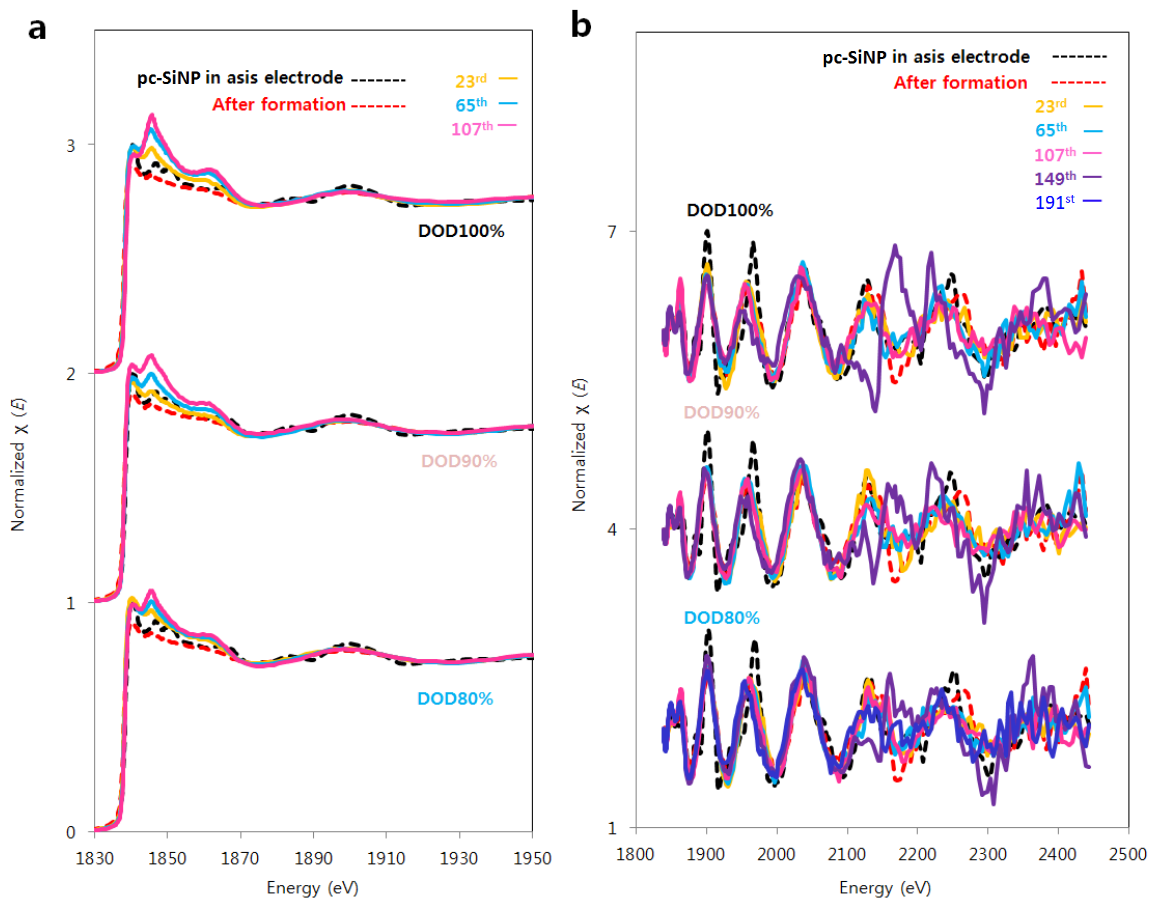

Supplementary Fig. 22

X-ray absorption near edge structure (XANES) and extended X-ray absorption fine structure (EXAFS) profiles at the Si K-edge for fully delithiated type-A electrodes cycled in 2032-type coin half-cells cycled under depth of discharge (DOD)80–100% over 190 cycles. **a** Stacked XANES profiles and (**b**) stacked EXAFS profiles. Black and red dotted lines are for electrodes before cycling and after the amorphization of polycrystalline Si nanoparticles, respectively. Prior to observation, the coin cells with type-A electrodes are cycled at 1 C to the target cycle number, and the electrode is fully delithiated by holding the potential at 1.5 V until the current decays to less than 0.001 C. The electrodes are washed using dimethyl carbonate (DMC) in Ar-filled glovebox, vacuum dried and transferred to the XAFS holder (see details in Methods under 'XAFS').

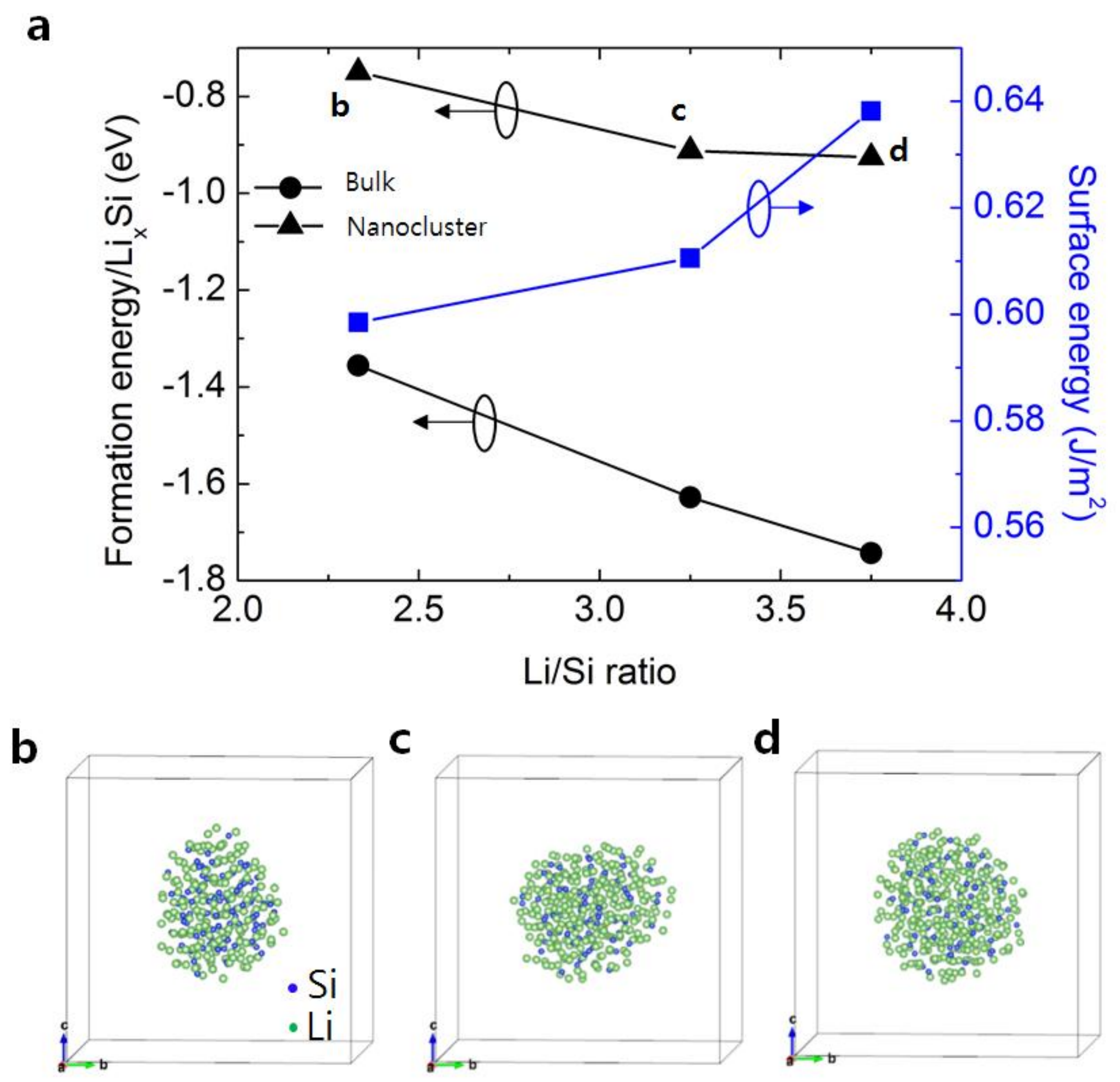

Supplementary Fig. 23

Density functional theory (DFT) calculation for formation/surface energy of Li–Si alloys with different sizes and Li concentrations. **a** Formation energy and surface energy (see details in Methods under 'Numerical calculations') as a function of fractional Li concentration (x = Li/Si) for bulk a-Li_xSi and ~2 nm Si nanoclusters for x = 2.33, 3.25, and 3.75. **b**–**d** Schematics of Li_xSi nanoclusters at x = 2.33, 3.25, and 3.75; blue and green spheres show Si and Li atoms, respectively. The final structures are obtained from classical molecular dynamics simulation implemented in the Lammps package with the reactive force field (ReaxFF).

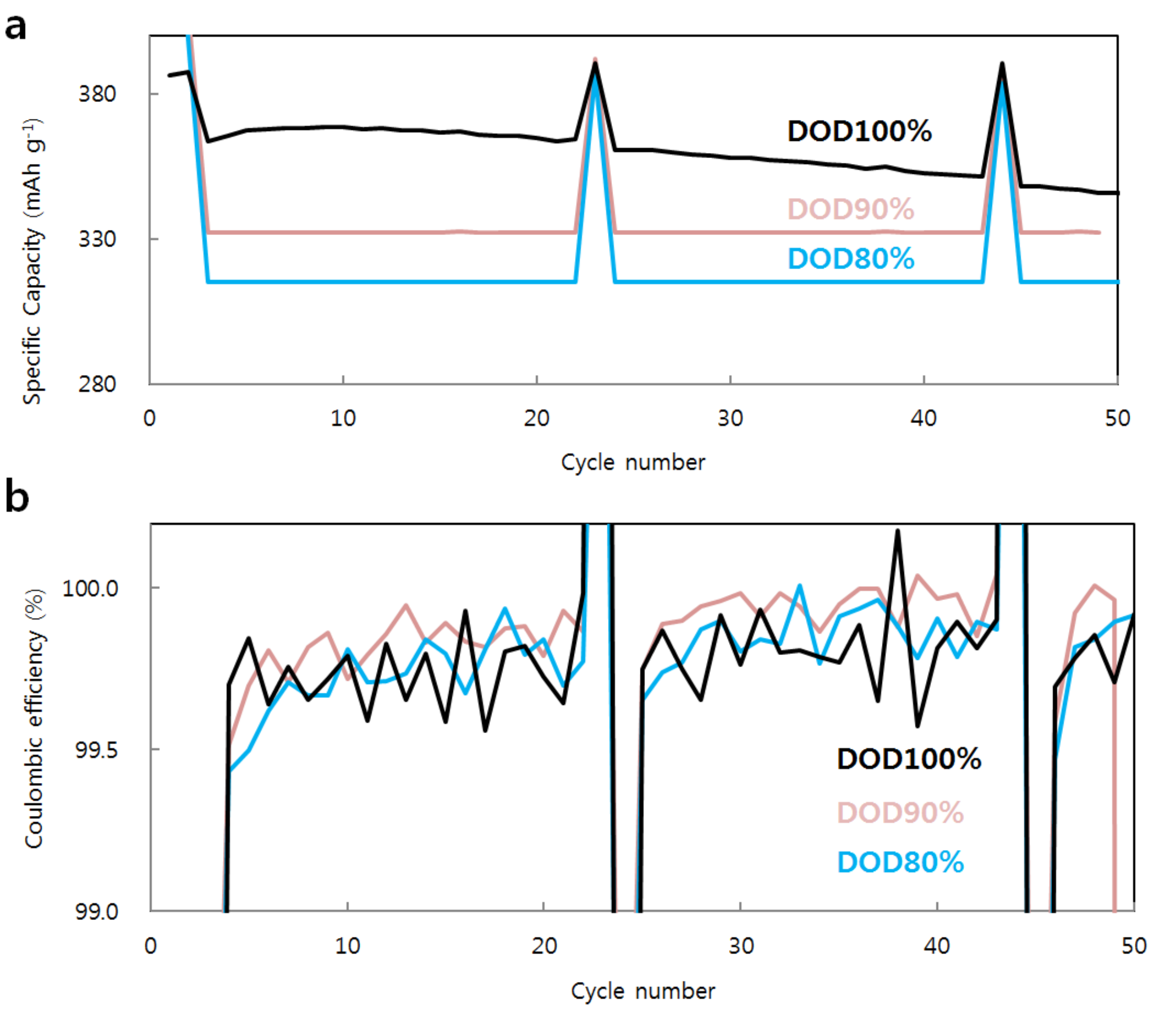

Supplementary Fig. 24

Coulombic efficiency (CE) of bare graphite (Gr) electrode. **a** Specific capacity and (**b**) CE of bare Gr electrodes in 2032-type coin half-cells cycled under different depth of discharge (DOD) controls. Black, pink, and blue lines correspond to the plots for DOD100%, 90%, and 80%, respectively.

Supplementary Table 1

A list of electrochemical control parameters (brown), electrochemical outputs (blue), and structural analysis methods (purple) used in this study. The corresponding figures are indicated in the right column. The experimental scheme based on the following list is also shown in Fig. 1.

**Electrochemical control parameter#**	**Inputs**	**Relevant figures**
**1**	**Depth of discharge (DOD)%: separating incremental *a*–*a* volume changes and a–c phase transformations**	**Fig.1–4,<br>SI-Fig.11–13**
**Electrochemical outputs#**	**Outputs**	**Relevant figures**
**1**	**Coulombic efficiency (CE)**	**Fig.4,<br>SI-Fig.11–13**
**2**	**Cumulative irreversible Li consumption**	**Fig.4 and SI-Fig.13**
**3**	**d*Q*/d*V***	**Fig.3, SI Fig.6,7**
**4**	**Symmetric EIS**	**SI-Fig.8,9**
**Structural analysis#**	**Analytical method**	**Relevant figures**
**1**	**TEM**	**Fig.5.6, SI-Fig.14–19<br>SI-Video.1,2**
**2**	**EELS/EDS**	**Fig.5, SI-Fig.17,19**
**3**	**XRD**	**Fig.6**
**4**	**NMR**	**Fig.7**
**5**	**XAFS**	**Fig.8, SI-Fig.22**
**6**	**XPS**	**SI-Fig.20**
**7**	**Electrode thickness**	**SI-Fig.21**
**8**	**DFT**	**SI-Fig.23**

Supplementary Table 2

Summary of new electrochemical findings in this work and the corresponding data source used for the interpretation.

	Electrochemical new findings	Interpreted from
**1**	**<u>An accelerated shift of electrochemical Li–Si reaction regimes by the repeated a–c phase transformations</u>**<br><br>The repeated a–c phase transformations accelerate the alteration of Li–Si electrochemical pathways from asymmetric to symmetric reaction sequences. In the former, c-$Li_{3.75(+\delta)}Si$ formed around ~50 mV asymmetrically transforms back into a-$Li_{<\sim 1.1}Si$ on delithiation, with the well-known large hysteresis at 430 mV. In the latter, c-$Li_{3.75(+\delta)}Si$ can reform a-$Li_{\sim 3.2\text{-}3.75}Si$ at as low as ~150 mV via a newly elucidated process, followed by the subsequent symmetric a–a transformations at 300 mV (a-$Li_{\sim 3.5}Si$→a-$Li_{\sim 2.0}Si$) and 550 mV (a-$Li_{\sim 2.0}Si$→a-Si). Repeating the a–c transformations under depth of discharge (DOD)100%, the regime shift occurs at around the 60th cycle, while it is postponed to after the 107th cycle when cycled under the lower DOD%.	**CE**<br>**d*Q*/d*V***
**2**	**<u>Quantitative and qualitative characterization of the evolving CE alterations by different Li–Si structural changes</u>**<br><br>Repeating the c-$Li_{3.75(+\delta)}Si$ formation/decomposition under DOD100%, typically featured as a capacity degradation factor, can increase CE up to ~99.9% in the most efficient manner among the given DOD controls, and minimize the cumulative irreversible Li consumption after incurring a certain amount of sacrificial Li consumption and capacity loss. This is quantitatively distinguished from that by mere amorphous Li–Si volume changes under DOD70–90%.	**CE**<br>**d*Q*/d*V***
**3**	**<u>Clarifying inherent nature of CE behaviours in different electrochemical Li–Si process regimes</u>**<br><br>When Si surface is fully exposed to the electrolyte, Coulombic efficiency (CE) has a strong correlation with the associated electrochemical regimes in the electrodes and the DOD controls. The asymmetric-to-symmetric shift can alter the susceptibility of CE. In the asymmetric regime, CE is more susceptible to the presence/absence of c-$Li_{3.75(+\delta)}Si$ upon (de)lithiation by the DOD controls. In contrast, in the symmetric regime, CE is more stabilized/saturated and higher in value regardless of the presence.	**CE**<br>**d*Q*/d*V***

Supplementary Table 3

Summary of mechanistic findings in this work, and the corresponding data source used for the interpretation.

	Mechanistic new findings	Interpreted from
**1**	<u>**An accelerated shift of Li–Si local environments' reaction sequences**</u><br>Repeated c-$Li_{3.75(+\delta)}Si$ formation/decomposition under depth of discharge (DOD)100% over cycles also accelerates the shift of reaction sequence of Li–Si local environments from asymmetric to symmetric. In the former, the environments hysterically interact among 6 different environments on (de)lithiation; isolated Si and overlithiated Si in c-$Li_{3.75(+\delta)}Si$ do not reform small Si clusters, and instead they hysterically form larger Si clusters and extended Si networks in a-$Li_{<\sim1.1}Si$ upon the quasi-two-phase process at 430 mV. In contrast, in the latter, only the 5 environments interact on (de)lithiation; there is no +δ components in c-$Li_{3.75(+\delta)}Si$, and isolated Si anions in c-$Li_{3.75}Si$ can reform Li-deficient isolated Si and small Si clusters at ~150 mV, followed by the double plateaus at 300 and 550 mV.	**d*Q*/d*V***<br>**NMR**<br>**XRD**
**2**	<u>**A shift from bulk- to surface-dominated systems**</u><br>The sequence shift in #1 is correlated with changes in $A_{(2Å\ Si–Si)}$ *. When $A_{(2Å\ Si–Si)}$ >~0.8–0.85, the sequence becomes asymmetric, and the Si morphology is bulkier, while when $A_{(2Å\ Si–Si)}$ <~0.8–0.85 the sequence is more symmetric and more surface-dominated, e.g. involving nano-structured Si smaller than 5 nm with a narrower feature size distribution. Also, when $A_{(2Å\ Si–Si)}$ >~0.8–0.85, Coulombic efficiency (CE) alteration is very susceptible to the presence/absence of c-$Li_{3.75(+\delta)}Si$, while when $A_{(2Å\ Si–Si)}$ <~0.8–0.85 CE is more stabilized and higher in value (~99.5–99.9%) regardless of c-$Li_{3.75(+\delta)}Si$ presence. This shift is accelerated by repeating the c-$Li_{3.75(+\delta)}Si$ formation/decomposition.<br>* $A_{(2Å\ Si–Si)}$ is the population index of Si–Si tetrahedral correlation obtained from XAFS analysis (see details in Methods under 'XAFS').	**CE**<br>**d*Q*/d*V***<br>**EIS**<br>**NMR**<br>**Electrode thickness**<br>**XPS**<br>**XAFS**
**3**	<u>**Interfacial stabilization in the surface-dominated system**</u><br>In the asymmetric-to-symmetric regime transition at $A_{(2Å\ Si–Si)}$ ~0.8–0.85, the +δ component in c-$Li_{3.75+\delta}Si$ (potentially reduction sources) disappears even under quasi-thermodynamic reactions. Also, the thickening of the electrode is saturated at the regime shift point. The same trend is observed in the FWHM of c-$Li_{3.75(+\delta)}Si$ XRD reflection. These trends indicate that in the symmetric regime, there are less destructive or more efficient stress release processes, with much more reversible reactions at the Si/SEI interface.	**CE**<br>**d*Q*/d*V***<br>**EIS**<br>**NMR**<br>**Electrode thickness**<br>**XPS**<br>**XAFS**
**4**	<u>**Altered surface energetics in sub-5 nm Li–Si structures**</u><br>Preliminary DFT calculation indicates that the driving force for lithiating a-Li_xSi beyond x = 3.25 in Si nanoclusters smaller than 5 nm is significantly lower compared with that in bulk Si, and that the surface formation energy significantly increases. In such a surface-dominated system, there may be less propensity for Li atoms to overlithiate the c-$Li_{3.75}Si$ nuclei inhomogeneously. Instead, breaking the residual Si–Si bonds is preferred, resulting in more uniform lithiation, i.e., the absence of +δ.	**d*Q*/d*V***<br>**DFT**<br>**XRD**<br>**NMR**<br>**Electrode thickness**

Supplementary Table 4

Physical parameters and composition ratios of materials in type-A and -B secondary particles.

	Properties	type-A	type-B
**Physical properties of polycrystalline nano-structured Si particles**	O_2 content (at%)	<0.5	
	Specific surface area (m^2 g^{-1})	17	
**Composition of secondary particles**	Ratio of nano-structured Si particles (wt%)	55.9	87.0
	Ratio of CNT (wt%)	7.8	10.9
	Ratio of Gr (wt%)	34.3	0
	Ratio of polyvinyl alcohol (wt%)	0.39	2.17
**Physical properties of secondary particles**	Specific surface area (m^2 g^{-1})	29.5	39.5
	Theoretical capacity (mAh g^{-1})	2273	3342
	Experimental first reversible capacity (mAh g^{-1})	2250	3350

Supplementary Table 5

Li–Si electrochemical processes associated with local structural changes. Using the notations in the main text, Si#d-X and Si#c-X denote the Xth Li–Si discharge (lithiation) and charge (delithiation) processes, respectively (see details in Methods under 'Reference electrochemistry'). The MAS ^{7}Li ss-NMR resonances are highlighted by blue (25–10 ppm, small Si clusters), yellow (10–0 ppm, larger Si clusters and extended Si networks and networks, including Si_5 ring and Si_3Si stars), pink (6–0 ppm, isolated Si^{4-} anions including c-$Li_{3.75}$Si), and green (0– -10 ppm, overlithiated crystalline phase, c-$Li_{3.75+\delta}$Si), respectively. Blue and red letters represent lithiation and delithiation processes, respectively. The Li–Si processes in the two electrochemical regimes as well as the ones in the 1st cycle are also indicated in the rightmost column: namely the 1st cycle and the asymmetric and symmetric regimes.

	Li–Si electrochemical processes	Li–Si electrochemical processes	Li–Si processes in ^{7}Li solid-state NMR spectrum	Li–Si processes in ^{7}Li solid-state NMR spectrum	Li–Si processes in ^{7}Li solid-state NMR spectrum	Li–Si processes in ^{7}Li solid-state NMR spectrum	Li–Si processes regime	Li–Si processes regime	Li–Si processes regime
			Li–Si local structure	Li–Si local structure	Li–Si local structure	Li–Si local structure			
Li/Li^{+} potential (mV) (lithiation /delithiation)	Li–Si processes	Stoichiometry	Small Si clusters	Large Si clusters and extended Si networks	Isolated Si	Overlithiated Si	1st cycle	Asymmetric regime	Symmetric regime
			20–10 ppm	10–0 ppm	10–0 ppm	-10 ppm			
100	Si#d-1 (only the 1st cycle)	c-Si > c-$Li_{3.75(+\delta)}$Si	✓	✓	✓	✓	↓		
250–300	Si#d-2	a-Si > a-$Li_{\sim 2.0}$Si		✓				↓	↓
100	Si#d-3	a-$Li_{\sim 2.0}$Si > a-$Li_{\sim 3.5-3.75}$Si	✓		✓			↓	↓
<50	Si#d-4	a-$Li_{\sim 3.75}$Si > c-$Li_{3.75+\delta}$Si (only in asymmetric reaction regime)			✓	✓		↓	
	Si#d-4 (broad)	a-$Li_{\sim 3.75}$Si > c-$Li_{3.75}$Si (only in symmetric reaction regime)			✓				↓
0–150	Si#c-1 (only in asymmetric reaction regime)	c-$Li_{3.75(+\delta)}$Si > c-$Li_{3.75(-\delta)}$Si			✓		↓	↓	
0–150	Si#c-1' (only in symmetric reaction regime)	c-$Li_{3.75}$S > a-$Li_{\sim 3.2-3.75}$Si	✓		✓				↓
300	Si#c-2	a-$Li_{\sim 3.5-3.75}$Si > a-$Li_{2.0}$Si	✓	✓					↓
430	Si#c-3	c-$Li_{3.75(-\delta)}$Si > a-$Li_{<\sim 1.1}$Si		✓			↓	↓	
>550	Si#c-4	a-$Li_{\sim 2.0}$Si > a-Si		✓			↓	↓	↓
