# Supplementary material for: Evolving affinity between Coulombic reversibility and hysteretic phase transformations in nano-structured silicon-based lithium-ion batteries
Source: Nat Commun. 2018 Feb 2;9:479. doi: 10.1038/s41467-018-02824-w (PMC5797158; doi:10.1038/s41467-018-02824-w)
Supplement: Supplementary file 3 — Description of Additional Supplementary Files [file 41467_2018_2824_MOESM3_ESM.pdf]

Description of Additional Supplementary Files

File Name: Supplementary Movie 1
Description: Movie clips of tilting STEM-HAADF image series for delithiated amorphous-Si in type-A electrode after the 2nd cycle.

File Name: Supplementary Movie 2
Description: Movie clips of close-up tilting STEM-HAADF image series for delithiated amorphous-Si in type-A electrode after the 2nd cycle.
